# Novel sequencing technologies and bioinformatic tools for deciphering the non-coding genome

**DOI:** 10.1515/medgen-2021-2072

**Published:** 2021-08-14

**Authors:** Jana Marie Schwarz, Richard Lüpken, Dominik Seelow, Birte Kehr

**Affiliations:** Department of Neuropediatrics, Charité–Universitätsmedizin Berlin, Corporate Member of Freie Universität Berlin and Humboldt-Universität zu Berlin, Berlin, Germany; NeuroCure Cluster of Excellence, Charité–Universitätsmedizin Berlin, Corporate Member of Freie Universität Berlin and Humboldt-Universität zu Berlin, Berlin, Germany; BIH–Junior Research Group Genome Informatics, Berlin Institute of Health at Charité-Universitätsmedizin Berlin, Berlin, Germany; BIH–Bioinformatics and Translational Genetics, Berlin Institute of Health at Charité-Universitätsmedizin Berlin, Berlin, Germany; Institute for Medical and Human Genetics, Charité–Universitätsmedizin Berlin, Corporate Member of Freie Universität Berlin and Humboldt-Universität zu Berlin, Berlin, Germany; Algorithmic Bioinformatics, Regensburg Center for Interventional Immunology (RCI), Franz-Josef-Strauß-Allee 11, 93053 Regensburg, Germany; University Regensburg, Regensburg, Germany

**Keywords:** whole genome sequencing, variant detection, structural variants, non-coding variants, bioinformatics

## Abstract

High-throughput sequencing techniques have significantly increased the molecular diagnosis rate for patients with monogenic disorders. This is primarily due to a substantially increased identification rate of disease mutations in the coding sequence, primarily SNVs and indels. Further progress is hampered by difficulties in the detection of structural variants and the interpretation of variants outside the coding sequence. In this review, we provide an overview about how novel sequencing techniques and state-of-the-art algorithms can be used to discover small and structural variants across the whole genome and introduce bioinformatic tools for the prediction of effects variants may have in the non-coding part of the genome.

## Introduction

High-throughput sequencing techniques have radically influenced our ability to obtain genomic information. These technologies provide large-scale datasets from whole exomes and whole genomes, resulting in a well-established and validated process for identifying small variants. The identification of larger, structural variants is improving with recent developments of new sequencing technologies and variant detection tools.

**Figure 1:**
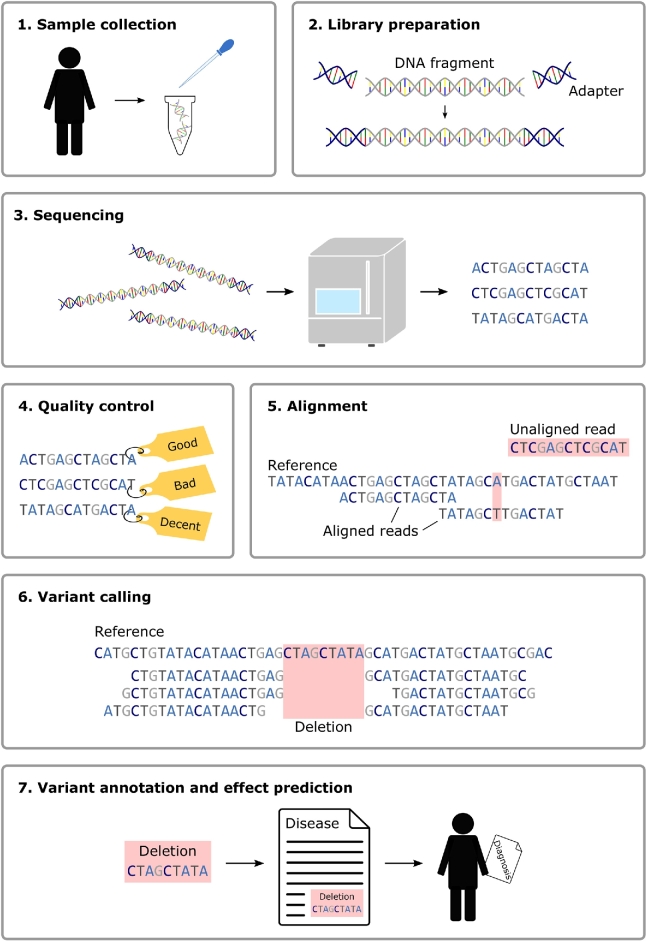
Standard steps in a sequencing and data analysis workflow.

The standard steps of a sequencing and data analysis workflow are illustrated in Figure 1. After sample collection and DNA extraction, a sequencing library that will be loaded onto a sequencing instrument is prepared. Modern sequencers produce vast numbers of short sequence pieces, termed reads. Before variants can be detected in the data, the reads need to be assigned to positions in a reference genome using a read alignment program. Variant detection and genotyping algorithms can then search for differences between the reads and the reference sequence. Finally, the impact of the variants on a phenotype is inferred through annotation and computational prediction of variant effects. Though the overall workflow is similar for whole exome and whole genome sequencing, the implementation of the individual steps can differ substantially depending on the chosen sequencing technology and variant type of interest.

Read alignment is the single most time consuming computational analysis step. Unless special hardware, e. g., field-programmable gate arrays (FPGA), specifically designed for the read alignment task is used [1], this step takes hours for whole genome data. Each of the millions of reads resulting from each genome needs to be compared to the roughly 3 billion base pairs of the human reference genome in order to find the sequence’s position of origin. It is essential to allow for small differences between read and reference in order to enable the alignment of reads containing variants or sequencing errors. All widely used alignment programs, e. g., BWA [2] for short read data and Minimap2 [3] for long read data, create an index of the reference genome that is comparable to the index in a textbook. The index allows a quick lookup of subsequences from the reads to identify candidate positions in the reference genome, eliminating the need to go through the entire reference genome for each read. These candidate positions are verified by a detailed read-to-reference comparison limited only to the respective part of the reference. Aligned reads are commonly stored in the binary alignment map (BAM) file format.

Accuracy and completeness of the alignment directly influence the performance of the following variant calling step. Variant calling includes variant detection and genotyping. Computational tools for variant calling implement entirely different algorithms depending on the variant type of interest and the sequencing technology used to generate the data. The calling of small variants, single-nucleotide variants (SNVs), and insertions and deletions of up to 50 bp (indels) is typically performed together. Variants larger than 50 bp, the SVs, require more elaborate algorithms. Most SV calling tools are specialized to recognize a single SV type, e. g., copy number variants (CNVs) or variants in a pre-defined size range.

**Table 1:** Whole genome sequencing technologies.

Company	Platform/protocol/flow cell type	Read length	Accuracy rating	Throughput per flow cell	Cost per coverage	Investment costs	SV detection	Phasing
Illumina	NovaSeq 6000 S4	+	+++	54–68 Gb/h	very low	high	+	o
Illumina	HiSeq X	+	+++	22–25 Gb/h	low	high	+	o
Illumina	HiSeq 4000	+	+++	15–18 Gb/h	low	medium	+	o
MGI	DNBSEQ-G400	+	+++	13.1 Gbp/h	very low	medium	+	o
MGI	DNBSEQ-T7	+	+++	250 Gbp/h	very low	high	+	o
Oxford Nanopore Technologies	PromethIon	+++	+	4.2 Gb/h	medium	medium	+++	+
Oxford Nanopore Technologies	GridIon	+++	+	0.7 Gb/h	high	low	+++	+
Oxford Nanopore Technologies	MinIon	+++	+	0.7 Gb/h	high	very low	+++	+
Pacific Biosciences	Sequel II HiFi	++	+++	1.5 Gb/h	very high	high	+++	+
Pacific Biosciences	Sequel II Long read	+++	+	6 Gb/h	high	high	++	++
10X Genomics	Chromium Linked Reads	+++	+++	N/A^1^	N/A^2^	medium	++	+++
MGI	stLFR	+++	+++	N/A^1^	low	very low	++	+++

^1^Throughput of linked read protocols depends on the short read platform used.

^2^10X Genomics discontinued the linked read protocol in 2020.

Variant calling is followed by variant annotation and interpretation. In general, variants are categorized based on either their effect on the DNA sequence or their functional effect on the gene product. The latter categorization is often used for coding variants, since the impact on the protein can often be directly deduced from the change of the DNA sequence. Categorization of variants located outside of protein coding genes is not so trivial, because less is known about these variants’ potential effects. Although examples of human disorders caused by the disturbance of non-coding regulatory elements have been identified, these are still a minority in comparison to the entirety of known disease mutations. This puts a challenge on currently available tools for variant interpretation.

This review provides an overview of state-of-the-art established and new whole genome sequencing technologies (Table 1), variant detection algorithms (Table 2), and variant evaluation tools (Table 3). We will first recapitulate whole genome sequencing technologies along with a summary of corresponding variant detection tools and then discuss computational approaches for variant interpretation.

## Detecting variation through whole genome sequencing

Short read sequencing is the most established and widely used technology for studying variation in whole genomes. Additional technologies that can reveal variants in regions of the genome that are inaccessible in short read data have emerged. In this section we discuss variant detection approaches for short read, long read, and linked read sequencing data and provide a brief overview of more specialized protocols for studying whole genomes.

### Short read whole genome sequencing

**Figure 2:**
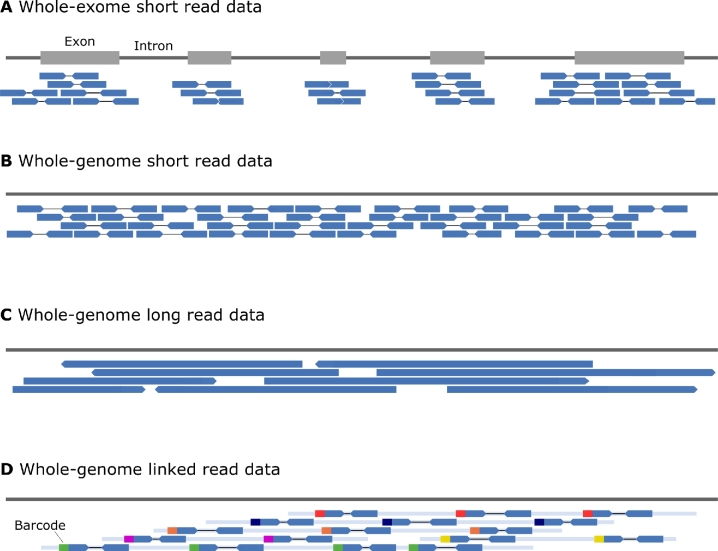
Whole exome and whole genome sequence data types.

Short read sequencing generates sequence reads between 100 and 300 bp in length at very low error rates (Figure 2B). In preparation for sequencing, the input DNA is sheared into fragments of about 300–600 bp in length. When using Illumina sequencing platforms, the DNA fragments are bridge amplified to form clonal clusters. When using MGI sequencing platforms, the fragments are converted into DNA nanoballs (DNBs). These processes allow the sequencing instruments to read a fixed number of base pairs, e. g., 150 bp, from both ends of the DNA fragments. The resulting sequences are output as read pairs, one read pair per fragment. The process is massively parallelized allowing the generation of up to 6 trillion base pairs (6 Tb) per sequencing run (Table 1). In order to reliably identify heterozygous variants, a typical aim is a minimum of 30-fold sequencing coverage of the genome. This translates to approximately 90 billion base pairs (90 Gb) or 600 million reads assuming a read length of 150 bp.

**Table 2:** Selection of variant calling tools.

Tool name	Variant types	Year of publication	Latest update	Source code	Reference
**Small variant callers for short read data**					
*GATK HaplotypeCaller*	SNVs, indels	2017	2021	https://github.com/broadinstitute/gatk	https://doi.org/10.1101/201178
FreeBayes	SNVs, indels	2012	2021	https://github.com/ekg/freebayes	https://arxiv.org/abs/1207.3907v2
GraphTyper	SNVs, indels	2017	2021	https://github.com/DecodeGenetics/graphtyper	PMID: 28945251
Samtools	SNVs, indels	2011	2021	https://github.com/samtools/samtools	PMID: 21903627
DeepVariant	SNVs, indels	2018	2020	https://github.com/google/deepvariant	PMID: 30247488
**Small variant callers for long read data**					
*Longshot*	SNVs	2019	2021	https://github.com/pjedge/longshot/	PMID: 31604920
Medaka	SNVs, indels	2017	2020	https://github.com/nanoporetech/medaka	unpublished
**SV callers for short read data**					
*Delly*	DEL, DUP, INV, TRL	2012	2021	https://github.com/dellytools/delly	PMID: 22962449
Lumpy/Smoove	DEL, DUP, INV, TRL	2014	2020	https://github.com/arq5x/lumpy-sv, https://github.com/brentp/smoove	PMID: 24970577
Manta	DEL, DUP, INV, TRL	2016	2019	https://github.com/Illumina/manta	PMID: 26647377
GRIDSS	Breakpoints	2017	2021	https://github.com/PapenfussLab/gridss	PMID: 29097403
GenomeSTRiP	CNV	2015		http://software.broadinstitute.org/software/genomestrip/	PMID: 25621458
BreakDancer	DEL, INV, TRL	2009	2015	https://github.com/genome/breakdancer	PMID: 19668202
Pindel	DEL, INV, TRL	2009	2017	https://github.com/genome/pindel	PMID: 19561018
*CNVnator*	CNV	2011	2020	https://github.com/abyzovlab/CNVnator	PMID: 21324876
*PopDel*	DEL	2021	2021	https://github.com/kehrlab/PopDel	https://doi.org/10.1038/s41467-020-20850-5
GraphTyper2	Only genotyping	2019	2021	https://github.com/DecodeGenetics/graphtyper	PMID: 31776332
MindTheGap	INS	2014	2020	https://github.com/GATB/MindTheGap	PMID: 25123898
PopIns	INS	2016	2020	http://github.com/bkehr/popins	PMID: 25926346
Pamir	INS	2017	2020	https://github.com/vpc-ccg/pamir/	PMID: 28881988
**SV callers for linked read data**					
*LongRanger*	DEL, INV, DUP	2019	2020	https://github.com/10XGenomics/longranger	PMID: 30894395
*NAIBR*	Breakpoints	2018	2019	https://github.com/raphael-group/NAIBR	PMID: 29112732
GROC-SVs	Breakpoints	2017	2020	https://github.com/grocsvs/grocsvs	PMID: 28714986
VALOR2	DEL, DUP, INV, TRL	2020	2020	https://github.com/BilkentCompGen/valor	PMID: 32192518
LinkedSV	DEL, DUP, INV, TRL	2019	2021	https://github.com/WGLab/LinkedSV	PMID: 31811119
**SV callers for long read data**					
Sniffles	DEL, DUP, INV, TRL, INS	2018	2020	https://github.com/fritzsedlazeck/Sniffles	PMID: 29713083
*SVIM*	DEL, DUP, INV, TRL, INS	2019	2021	https://github.com/eldariont/svim	PMID: 30668829
NanoSV	DEL, DUP, INV, INS	2017	2019	https://github.com/mroosmalen/nanosv	PMID: 29109544
PBSV	DEL, DUP, INV, TRL, INS		2020	https://github.com/PacificBiosciences/pbsv	unpublished
npInv	INV	2018	2020	https://github.com/haojingshao/npInv	PMID: 30001702
cuteSV	DEL, DUP, INV, TRL, INS	2020	2021	https://github.com/tjiangHIT/cuteSV	PMID: 32746918

Tools referenced in the main text are in italics. DEL, deletions. DUP, duplications. INV, inversions. TRL, translocations. CNV, copy number variants.

Short read data are well suited for detecting and genotyping SNVs and indels across the whole genome. Approaches that compare the read sequences to the linear reference genome, e. g., GATK HaplotypeCaller [4], are widely used. In contrast to small variants, SVs are often too large to be spanned by a single short read. By looking for certain data signatures, SVs can be indirectly detected in short reads. CNVs result in changes in the number of read pairs covering the deleted or duplicated part of the genome, e. g., detected by CNVnator [5]. Breakpoints of any type of SV are visible in the alignment as changes in the alignment distance of the two reads in a pair, e. g., detected by Delly [6] and PopDel [7], and as split reads, where the beginning of a read aligns to a different region of the genome than the end of the same read. A recent benchmark study [8] assessed SV detection approaches for short read data and demonstrated differences in their strengths.

The high accuracy of short read data allows for very reliable genotyping of SNVs and indels. In addition, short reads can reveal thousands of SVs per genome. Short read data are, however, inherently limited in repetitive regions of the genome. As SVs often occur in repetitive sequences, e. g., as variable numbers of tandem repeats or flanked by repeat sequences, this limitation clearly affects our ability to use short read data to detect SVs. We can overcome this problem by using long read sequencing data. This approach detects several times more SVs [9], [10] and identifies small variants in genomic regions inaccessible with short read data.

### Long read whole genome sequencing

Modern long read technologies produce reads of approximately 10–100 kb length from preferably unfragmented DNA (Figure 2C). The Pacific Biosciences (PacBio) technology relies on the synthesis of a complementary DNA strand by a single polymerase enzyme emitting light when incorporating a nucleotide to the growing DNA strand [11]. The sequencing instruments from Oxford Nanopore Technologies pass a single-stranded DNA molecule through a protein pore and measure an ionic current corresponding to the nucleotides residing in the pore at each moment [12]. Though these processes are massively parallelized, they do not yet reach the throughput and low cost of short read sequencing (Table 1). Despite enormous improvements in accuracy, long read data still suffer from much higher error rates than short read data. To circumvent the lower accuracy, Pacific Biosciences has launched a high-fidelity (HiFi) sequencing protocol, which sequences the same DNA fragments several times to allow computing a consensus sequence, thereby averaging out many sequencing errors. A limitation of PacBio sequencing is the high amount of DNA required as input.

Though long reads are less suited to detect SNVs and indels because of their low per base accuracy, the increasing availability and dropping error rate of long read data has led to the development of the first dedicated long read SNV detection tools, e. g., Longshot. In contrast, the long read length simplifies the identification of SVs, e. g., using SVIM [13], as a single long read often spans an entire SV. Most notably in repetitive sequences, long read methods outperform short read methods when searching for SVs. Because long reads frequently span multiple variants, detected variants can be phased into the parental haplotypes, e. g., using WhatsHap [14]. Despite these advantages, the high cost and high error rate are still barriers to a widespread use of long read sequencing.

### Linked read whole genome sequencing

Linked reads combine some of the strengths of short and long read sequencing by combining information from longer DNA molecules with high accuracy and lower costs (Figure 2D). Technically, linked read sequencing is a specialized protocol for short read sequencing. Long DNA molecules are isolated from a sample. During sequencing library preparation, the long molecules are sheared into shorter fragments in a process that ligates barcodes to all fragments. All short fragments resulting from the same original long DNA molecule are labeled with the same barcode. The Chromium platform by 10X Genomics achieved this by separating long molecules within oil droplets from each other whereas the stLFR protocol marketed by MGI uses microbeads and transposons for fragmentation and barcoding. Finally, the barcoded fragments are sequenced with standard short read sequencing technologies. The barcode is sequenced as part of each read pair. Reads with the same barcode typically originate either from the same long molecule or from a small set of long molecules.

Linked reads are, at their core, short reads. This means that linked read sequencing achieves the same order of throughput and high accuracy as short read sequencing and the data are well suited for detecting small variants. In addition, linked reads contain long range sequence information included in the barcode sequences. Barcodes can resolve ambiguity in read alignment or can be used to phase variants to parental haplotypes, e. g., using LongRanger [15]. The long range information is useful to resolve SVs, e. g., using NAIBR [16], and may even be used for local assemblies. Some repeat elements inaccessible to standard short read data, such as mobile elements, can be resolved with linked reads. Still, many other repeats remain difficult to handle and may only be resolvable by actual long reads or further technological development. An additional disadvantage is that bioinformatics tools to analyze linked read data lag behind those for short and long read data. This is why linked reads have not been widely adopted despite their great potential.

### Specialized whole genome protocols

A variety of additional technologies for analyzing whole genomes are available. Optical genome mapping using the Bionano Saphyr system, also called whole genome imaging, can examine ultra-long DNA molecules of hundreds of kilobases in length, though it does not read the full sequence. The long DNA molecules are fluorescently labeled at specific 6-bp sequence motifs. In thousands of nanochannels, the labeled molecules are linearized and imaged, and the distances between motif occurrences are detected. This generates a footprint of the sequence, allowing reliable detection of large SVs. Though the average length of assayed molecules surpasses that of long and linked read sequencing technologies, the resolution is low compared to that of sequencing, meaning that the technology does not reveal small variants [17].

Single-cell DNA sequencing protocols provide data from whole genomes of individual cells. This is widely used in research on somatic variation in cancer genomes and when studying cancer genome heterogeneity. Specialized single-cell protocols can reveal variation that is otherwise hidden. For example, the Strand-Seq protocol can detect inversions, including those that are flanked by very long repeats. Prior to sequencing, one of the two DNA strands is digested in each cell, resulting in sequences from only one strand. The inverted sequence becomes almost trivially visible in the alignment of the reads to the reference genome. Major drawbacks of Strand-Seq are that cell lines are needed as input material and that the library preparation is comparably sophisticated.

Other methods, such as Hi-C, ChiP-Seq, and ATAC-Seq, provide functional and sequence information across the whole genome, yet play a less important role for variant calling. These technologies are reviewed by Guo et al. (this edition).

## Variant interpretation

Significant progress has been made in predicting the deleteriousness of non-synonymous variants in the coding genome, e. g., with Polyphen [18] or SIFT [19], over the course of the last two decades. MutationTaster [20] was the first tool that allowed the analysis of non-coding variants, though restricted to those within protein coding genes. Current tools reach prediction accuracies of about 90 % in artificial benchmarking settings. Results improve substantially in regular usage when known polymorphisms are excluded from the analysis. The chance of identifying disease mutations is further improved by including phenotype information through software such as MutationDistiller [21] because this limits the analysis to variants located in promising candidate genes. Computer-based analysis of facial dysmorphologies, e. g., with Face2Gene,1https://www.face2gene.com/ (FDNA Inc., Boston, MA, USA). may also help to highlight genomic regions of interest. Other tools, e. g., VarFish [22], allow for family trio analysis, excluding additional variants and detecting *de novo* mutations. In spite of recent progress, the diagnostic rates for whole exome sequencing (WES) usually remain below 50 % [23]. It is clear that the low hanging fruit of “easy-to-solve” monogenic diseases has already been picked. The remaining cases may be caused by SVs, which are notoriously hard to detect by WES, more subtle (deep) intronic variants affecting splicing [24] (Krude et al. this edition), or variants altering gene regulation. The latter are frequently located outside of the coding sequence, often hundreds of bases away from the gene they act on [25], [26]. Focusing the search for disease mutations on the extragenic space has enormous potential to reveal the molecular basis of currently undiagnosed genetic diseases. Yet less than 1 % of the disease mutations listed in the ClinVar database [27] are located outside of protein coding genes (Krude et al. this edition).

While whole genome sequencing can reveal such variants, it is challenging to evaluate their effect. The two main obstacles are the low number of known extragenic disease mutations and the lack of knowledge about their pathomechanisms. Both are needed to develop automatic prediction tools, which can assess the potential effects of non-coding variants. Regardless of these limitations, several tools have been developed that take the current knowledge into account, e. g., the Genomiser [28], CADD [29], or RegulationSpotter [30] (Table 3). Different approaches can also be combined as reflected by meta-tools, which merge the results from other predictors, such as Ensembl VEP [31] or SNPnexus [32].

**Table 3:** Selection of tools for variant interpretation with characteristics and limitations.

Name	Main purpose	Availability	Variant types	Prediction	Annotation	Graphical output	Prediction/annotation based on	Limitations	Requires extended knowledge in bioinformatics	URL	Reference
NCBoost	predict pathogenicity of variants	**download:** precomputed scores	SNV	X	–	–	conservation features, gene features, sequence context features	precomputed scores only available for selected variants	X	https://github.com/RausellLab/NCBoost	PMID: 30744685
CADD	predict deleteriousness of variants	**download:** precomputed scores for selected variants **web-interface:** offers precomputed scores for selected variants or VCF upload	SNV, selected indels	X	–	X	chromatin/epigenetic features, conservation features, gene features, sequence context features	web-interface service restricted to VCF file with maximum 100,000 variants and 2 MB	(X)	https://cadd.gs.washington.edu/	PMID: 30371827
DeepSEA	predict the chromatin effects of sequence alterations	**download:** complete software for local installation **web-interface:** VCF upload	SNV, indel	X	–	X	chromatin/epigenetic features, conservation features	web-interface service recommended only for VCF files with < 50,000 variants	(X)	http://deepsea.princeton.edu/	PMID: 26301843
Genomiser	identify regulatory variants in Mendelian diseases	**download:** precomputed scores or complete software for local installation	SNV, indel	X	–	–	chromatin/epigenetic features, conservation features, population/frequency features, sequence context features	only local installation	X	https://charite.github.io/software-remm-score.html	PMID: 27569544
RegulationSpotter	annotation and functional prioritization of variants	**web-interface:** analysis of single variants or VCF files	SNV, indel	–	X	X	chromatin/epigenetic features, conservation features, gene features, population/frequency features	no local installation	–	https://www.regulationspotter.org/	PMID: 31106382
RegulomeDB	regulatory annotation of variants or genomic regions	**web-interface:** analysis of known variants with dbSNP IDs	SNV, indel	–	X	X	chromatin/epigenetic features, integrates DeepSEA	analysis restricted to known dbSNP IDs	–	https://regulomedb.org/regulome-search	PMID: 31228310
SNPnexus	assist in functional prioritization of variants	**web-interface:** analysis of single variants or VCF files	SNV, indel	–	X	X	conservation features, epigenetic features, gene features, phenotype/disease features, population/frequency features, integrates CADD, DeepSEA, ReMM, and others	web-interface service restricted to VCF file with maximum 100,000 variants	–	https://www.snp-nexus.org/v4/	PMID: 32496546
VEP	determine effects of variants on genes, transcripts, protein sequences, and regulatory regions	**download:** complete software for local installation **web-interface:** analysis of single variants or VCF files	SNV, indel, CNV, SV	–	X	X	chromatin/epigenetic features, conservation features, gene features, population featuressequence features, integrates CADD and others	upload limited to 50 Mb	–	https://www.ensembl.org/info/docs/tools/vep/index.html	PMID: 27268795
SVscore	assess deleteriousness of SVs	**download:** complete software for local installation	SV	X	–	–	gene features, integrates CADD	only local installation	X	https://github.com/lganel/SVScore	PMID: 28031184
TAD fusion score	quantify potential of large deletions to disrupt 3D genome structure	**download:** complete software for local installation	SV (CNV, esp. DEL)	X	–	–	chromatin/epigenetic features, TAD boundaries	only local installation	X	https://github.com/HormozdiariLab/TAD-fusion-score	PMID: 30898144
TADA	functional annotation and pathogenicity prediction of CNVs	**download:** complete software for local installation	SV (CNV, i. e., DEL and DUP)	X	X	–	chromatin/epigenetic features, conservation features, gene features, TAD boundaries	only local installation	X	https://github.com/jakob-he/TADA	https://doi.org/10.1101/2020.06.30.180711v1
SVFX	predict pathogenicity of SVs	**download:** complete software for local installation	SV	X	–	–	chromatin/epigenetic features, conservation features, TAD boundaries	focuses on somatic SVs in cancer and germline SVs in common diseases	X	https://github.com/gersteinlab/SVFX	PMID: 33168059
StrVCTVRE	predict pathogenicity of SVs	**download**: complete software for local installation	SV	X	–	–	conservation features, expression features, gene features	only SVs of DUP and DEL type, which are overlapping an exon are analyzed	X	https://github.com/andrewSharo/StrVCTVRE	https://doi.org/10.1101/2020.05.15.097048v3

X = applies; (X) = partly applies; – = does not apply. DEL, deletion. DUP, duplication. VCF, variant call format.

The evaluation of all extragenic variants often begins with annotation of their location. For each variant, information is gathered whether it is located in a known regulatory region and whether this region is conserved during evolution. Exploiting information about phylogenetic conservation is a common and well-established method used by many tools (Table 3). The rationale is that strongly conserved positions are of high functional importance and disruption through variation in the DNA sequence is considered potentially pathogenic.

The next step after annotation is evaluation of functional impact. Some tools use the chromatin state where the variant is located to determine if a variant could interfere with gene expression. Chromatin state data come from public datasets on histone marks, transcription factor binding sites (TFBSs), DNase I hypersensitive sites (DHS), and long range genomic interactions (topologically associating domain [TAD] boundaries) in different cell types or tissues. Many data are generated and curated by large consortia, such as BluePrint [33], ENCODE [34], FANTOM5 [35], or Roadmap Epigenomics [36]. The idea that a variant residing in a putative promoter region, as indicated, e. g., by DNase I hypersensitivity, characteristic histone modifications, and TFBSs, impacts gene expression is appealing. Though the amount of available data is increasing, our incomplete understanding of exactly how small variants, such as SNVs and indels, affect gene regulation hampers the identification of disease mutations at present (see also Guo et al. this edition).

It has been shown, for example, that enhancers often act redundantly with other enhancers serving as a backup and even the deletion of a complete enhancer does not automatically have an impact on the expression of the regulated gene [37]. This makes it extremely difficult to predict if and how a more subtle deletion or exchange of a single nucleotide within an enhancer might affect gene expression. The same applies to TFBSs, where it is often unclear whether or not a specific SNV has a profound effect on transcription factor binding. In other words, there is no simple correlation between binding score and binding affinity. To complicate matters further, a reduced binding affinity does not necessarily result in disease, as transcription levels may still be sufficient to ensure normal protein amounts.

The lack of knowledge about molecular pathomechanisms of small, putative regulatory variants is mirrored in a lack of training data for developing prediction algorithms. Different approaches have been developed to circumvent this shortcoming. Instead of using the small number of published “real” disease mutations in the non-coding genome, the authors of CADD simulated a set of “proxy-neutral” *versus* “proxy-deleterious” variants that were categorized based on whether or not they had been a target of purifying selection. While the “proxy-neutral” variants were real variants which persisted for millions of years without being selected against, the “proxy-deleterious” set consisted of artificial variants without selective pressure. This also reduced the bias towards conservation in the group of “proxy-deleterious” variants, which would have been introduced by using known disease mutations. Other authors, e. g., those of the Genomiser [28] or StrVCTVRE [38], manually curated small, high-quality datasets of known regulatory or structural variants for the training of their tools. Due to the low number of newly discovered extragenic disease mutations as prospective controls, it is currently hard to say which approach will lead to more robust results.

Though the size of SVs suggests that their effect on gene expression is more straightforward to evaluate, this is currently not the case. Predicting the effects of SVs differs from the evaluation of small variants in one central aspect: SVs may completely delete one or multiple genes or crucial parts of genes, or disturb TADs (Krude et al. this edition, Guo et al. this edition). Apart from these dramatic consequences, the following information, amongst others, is relevant for and has been implemented in available tools (Table 3): **(i)** locations of transcription start sites and alternative splice sites, **(ii)** differential gene expression, **(iii)** epigenetic information about chromatin state as conferred by DNA methylation and histone modifications, and **(iv)** the presence of TFBSs. Because only a low number of disease causing SVs are known, a robust estimate of the predictive performance of the available tools is difficult.

In summary, current software can fairly reliably identify genomic regions likely to play a role in gene regulation. Improving the prediction of the functional impact of the DNA variants is subject of future research.

## Current limitations and outlook

Two decades after the release of the human reference genome in 2001 [39], [40] and the official completion of the Human Genome Project in 2003, parts of the genomic sequence still remain unknown [41]. The latest genome version GRCh38 lacks approximately 5 % of the sequence, mainly heterochromatic, highly repetitive regions, which are hard to sequence and map. The international “Telomere-to-Telomere” (T2T) consortium aims to close this gap using new sequencing technologies, such as ultra-long read nanopore whole genome sequencing. As proof of principle, the complete sequence from telomere to telomere of a human X chromosome was published last year [42]. A preliminary version of a complete female genome (46, XX), essentially lacking only the information for encoded rRNA, is already freely available to the scientific community.2https://github.com/nanopore-wgs-consortium/CHM13#telomere-to-telomere-consortium Another initiative, the “Genome In A Bottle” (GIAB) consortium, strives to provide validated benchmark datasets and best-practice protocols for detection of small and large variants [43], [44]. These are still limited to certain regions of the genome.

Though these efforts shed light on the sequence itself, our understanding of how variation in the non-coding sequence impacts function is very incomplete. We are only just beginning to understand the role of non-coding DNA and the interaction of DNA with its environment, e. g., through histone modifications. High-throughput techniques, such as ChIP-seq and its variations, and genome architecture studies (Guo et al. this edition) will lead to novel insights into the number and the functional relevance of the non-coding parts. Other approaches using saturation or random mutagenesis of genomic elements, e. g., massively parallel reporter assays (MPRAs) [45], or mutagenesis within short PCR fragments, including TFBSs as performed in our research unit, will reveal the effects single-nucleotide variants or short indels have on the function of these elements.

Still, since so many different players – DNA variants, histone modifications, distant enhancers, and cell-specific proteomes, to name just a few – are involved in gene regulation, this field will remain challenging for the next decades. Non-coding is non-trivial.
